# P-2000. A Case-Control Study of the Risk of COVID-19 Infection in Underserved Persons with HIV (PWH) in Middle Tennessee

**DOI:** 10.1093/ofid/ofae631.2157

**Published:** 2025-01-29

**Authors:** Mallika Rao, Derek Wilus, Vladimir Berthaud

**Affiliations:** Meharry Medical College, Nashville, Tennessee; Meharry Medical College, Nashville, Tennessee; MEHARRY MEDICAL COLLEGE, NASHVILLE, Tennessee

## Abstract

**Background:**

The COVID-19 pandemic had disproportionately affected racial and ethnic minorities. We undertook this study to examine the risk factors for COVID-19 infection and death in African Americans with HIV.

**Methods:**

We conducted a prospective case-control study to assess the risk for COVID-19 infection in a clinic serving predominantly underserved African Americans in Middle Tennessee. Matched cases and controls were tested for COVID-19 by PCR around the same time. COVID-19 information, demographics, clinical and social determinants of health (SDoH) data were collected from electronic health records. Chi-square and Fisher’s exact tests were applied to evaluate the associations between outcome and predictor variables. Binary logistic regression was used to examine the effects of 18 predictors on COVID-19 infection. R/RStudio version 4.3.2 was used. The local IRB approved the study protocol.

**Results:**

From 4/2020 to 11/2023, 91 PWH tested COVID-19-positive and 181 negative. We excluded 11 cases and 29 controls due to missing SDoH data; 81.0% of the patients were African American, 26.3% were female, and 46.1% fell into the 45-64-year-old age group. Among 73 symptomatic cases, the most common symptoms were cough, fever, and chills. 28/91 were admitted to the hospital, 11/28 went to ICU, and 8/28 died. All the deceased had >1 cardiovascular risk factor. 12/91 cases had a CD4 cell count < 200/mm3 and 73/91 had an HIV-1 RNA level < 200 copies/mL. In the bivariate analysis, incarceration (p < 0.001), renal failure (p < 0.001), smoking (p < 0.001), alcoholism (p = 0.003), drug use (p = 0.023), and cardiovascular risk (p = 0.023) were highly associated with COVID-19 infection. In the multivariable model, the most strongly associated risks were incarceration [OR = 18.68; 95% CI: (4.66, 74.87); p < 0.001], renal failure [OR = 40.62; 95% CI: (3.96, 416.83); p = 0.002], hyperlipidemia [OR = 3.94; 95% CI: (1.54, 10.06); p = 0.004], 25-44 years of age [OR = 2.48; 95% CI: (1.10, 5.58); p = 0.028], no insurance [OR = 0.36; 95% CI: (0.14, 0.92); p = 0.033], and smoking [OR = 2.20; 95% CI: (1.01, 4.82); p = 0.048].

**Conclusion:**

Incarceration, renal failure, and cardiovascular disease were very strong risk factors for COVID-19 infection in this population of PWH in Middle Tennessee.

**Disclosures:**

Vladimir Berthaud, MD MPH, GILEAD SCIENCES: Grant/Research Support|GSKVIIV: Grant/Research Support|MODERNA: Grant/Research Support|NOVAVAX: Grant/Research Support

